# Sex-biased gene expression in the brown alga *Fucus vesiculosus*

**DOI:** 10.1186/1471-2164-14-294

**Published:** 2013-05-01

**Authors:** Maria João F Martins, Catarina F Mota, Gareth A Pearson

**Affiliations:** 1 CCMAR,CIMAR-Laboratório Associado, Universidade do Algarve, Gambelas, Faro 8005-139, Portugal

**Keywords:** Brown algae, *Fucus*, Heterogametic sexes, Reproduction, Sex-biased genes, Sperm, Transcriptomics

## Abstract

**Background:**

The fucoid brown algae (Heterokontophyta, Phaeophyceae) are increasingly the focus of ecological genetics, biodiversity, biogeography and speciation research. The molecular genetics underlying mating system variation, where repeated dioecious – hermaphrodite switches during evolution are recognized, and the molecular evolution of sex-related genes are key questions currently hampered by a lack of genomic information. We therefore undertook a comparative analysis of male and female reproductive tissue transcriptomes against a vegetative background during natural reproductive cycles in *Fucus vesiculosus*.

**Results:**

Over 300 k reads were assembled and annotated against public protein databases including a brown alga. Compared with the vegetative tissue, photosynthetic and carbohydrate metabolism pathways were under-expressed, particularly in male tissue, while several pathways involved in genetic information processing and replication were over-expressed. Estimates of sex-biased gene (SBG) expression were higher for male (14% of annotated orthologues) than female tissue (9%) relative to the vegetative background. Mean expression levels and variance were also greater in male- than female-biased genes. Major female-biased genes were carbohydrate-modifying enzymes with likely roles in zygote cell wall biogenesis and/or modification. Male-biased genes reflected distinct sperm development and function, and orthologues for signal perception (a phototropin), transduction (several kinases), and putatively flagella-localized proteins (including candidate gamete-recognition proteins) were uniquely expressed in males. Overall, the results suggest constraint on female-biased genes (possible pleiotropy), and less constrained male-biased genes, mostly associated with sperm-specific functions.

**Conclusions:**

Our results support the growing contention that males possess a large array of genes regulating male fitness, broadly supporting findings in evolutionarily distant heterogametic animal models. This work identifies an annotated set of *F. vesiculosus* gene products that potentially regulate sexual reproduction and may contribute to prezygotic isolation, one essential step towards developing tools for a functional understanding of species isolation and differentiation.

## Background

The evolution of sexual reproduction and sexual dimorphism has stimulated numerous studies at the molecular, genetic, ecological and behavioural levels [1-7]. Nevertheless, detailed molecular knowledge of the processes involved, and their role in delimiting species boundaries remains limited [6,8]. Genes that mediate sexual reproduction often evolve rapidly [2,9], contributing to prezygotic isolation and speciation [9-11]. However, in the early stages of reproductive isolation, ecological divergence and niche-partitioning may contribute importantly to speciation without the direct involvement of genetic isolating mechanisms [8,10,12,13].

Recent advances in high throughput transcriptomics now make possible the identification of genes that are differentially expressed in males and females (i.e. sex-biased genes, SBG), and inferences concerning the major evolutionary forces involved [4]. Two main patterns have emerged from studies largely focused on metazoans. First, genes contributing to reproductive isolation are fast-evolving, often driven by positive selection [14]. Second, SBG expression is widespread within the genome [4]. SBG are therefore likely to play an important role in reproduction, and thus be subjected to powerful selection (e.g. for species recognition, sexual selection; [9]). Interestingly, male-biased genes are generally more numerous than female-biased genes, more highly expressed on average, and often show evidence of higher divergence rates between species ([4]; but see [15,16]). Explanations for these phenomena usually focus on functional pleiotropy (i.e. additional non-reproductive roles) for female-biased genes, although intense male-male competition, or greater transcriptional activity in male germ tissue have also been suggested [17-19].

Brown algae (Heterokontophyta) are an independent multicellular lineage [20], and therefore an attractive model for reproductive and sexual evolution from a comparative genomic viewpoint. Fucoid brown seaweeds are oogamous broadcast spawning marine taxa that dominate temperate intertidal shores, with key ecological roles as ecosystem engineers and primary producers. The intertidal genus *Fucus* provides a particularly interesting system for studies of sexual dimorphism and reproductive isolation: recent diversification in the Atlantic within the last 5 My [21] resulted in the evolution of well-separated clades, but also more recently diverged species and cryptic entities capable of hybridization [22-25]. Moreover, the mating system in fucoids is labile, and has undergone at least two independent transitions between dioecy and hermaphroditism within *Fucus*[21]. In externally fertilizing taxa, reproductive isolation may arise through both differential temporal and spatial timing of gamete release controlling prezygotic isolation [26-30], and the evolution of gametic incompability mechanisms [9,31,32]. The evolution of molecules involved in gamete-gamete recognition in free-spawning invertebrates have been widely studied, and rapidly-evolving proteins on gamete surfaces are of major importance in species recognition and speciation [9,32]. Egg and sperm proteins may be engaged in an arms race driven by several processes, from sexual conflict and sperm competition to reinforcement, frequency-dependent selection, as well as ecological pressures [9], contributing to the build-up of post-mating prezygotic barriers and divergence leading to speciation.

Previous work has established basic cues and physiological mechanisms behind spawning synchrony in fucoids (e.g*.*[33-37]). Fucoid oogametes are released as gametangia from the reproductive tissue (receptacles) into the surrounding seawater on strictly semilunar cycles in marine intertidal environments [38-40], on favourable daytime low tides (approximately two-week intervals [30,37]), with interspecific variation in the daily pattern of gamete release recognized [29,30]. Similarly, earlier work has established the basic characteristics of gamete-gamete recognition in fucoids [41,42]. However, further progress in understanding variation underlying species differences has been hampered by the lack of available genomic information. The main aim of this study was to compare reproductive and vegetative transcriptomes in a natural population of male and female *F. vesiculosus*. The potential function of annotated ESTs showing sex-biased expression is discussed, with particular reference to differentially expressed metabolic pathways, signalling, and proteins potentially involved in gamete-gamete interactions. We found evidence for male-biased expression in *F. vesiculosus* related mainly to sperm-specific functions. Evidence for possible pleiotropy in the female is discussed.

## Methods

### Plant collection, RNA extraction, cDNA synthesis and pyrosequencing

A total of 20 adult reproductive *F. vesiculosus* (10 × males and 10 × females) per sampling date were collected in 2008 at Praia Norte, Viana do Castelo, Portugal (41°41′59″N; 8°51′19″W). Sampling was carried out beginning at sunrise on Sep 30 and Oct 9, corresponding to spring (S) and neap (N) tide phases, respectively. Sampling was performed in this way to increase transcript coverage during the semilunar reproductive cycles of gamete maturation and release. A receptacle (reproductive tissue; see Additional file 1) was taken from each individual and transverse sections were examined under a field microscope (40 × magnification) to identify and confirm the sexual phenotype. The remaining mature receptacles (separate pools of males and females) were briefly washed in seawater, wiped to remove surface epiphytes, and then flash-frozen in liquid nitrogen for transport to the laboratory. Vegetative tips (pooled from male and female algae) were treated in the same way. In the laboratory, tissues were lyophilized prior to RNA extraction following [43]. RNA was digested with RNase-free DNase (QIAGEN) for 15 minutes at room temperature and then purified with the RNeasy MIDI kit (ca. 1 mg total RNA; Qiagen). RNA concentration was estimated by spectrophotometry (GeneQuant, GE Healthcare); integrity was confirmed by running samples on a 1.2% agarose gel.

Poly-A mRNA was isolated from total RNA (ca. 1 mg) using the Oligotex mRNA Midi kit (Qiagen). Double stranded cDNA was constructed using the SuperScript^®^ One-Cycle cDNA kit (Invitrogen), following the manufacturer’s instructions. Four independent reactions were carried out for each of the six library samples: two using poly dT priming primers (Oligo d(T)_25_ VN) and two reactions using random primers (N_15_). Double-stranded cDNA syntheses were purified using CyScribe GFX Purification Kit (GE Healthcare). A fluorometer was used to estimate DNA concentration (Picofluor, Turner Biosystems). The resulting cDNA (2–3 ug) was adjusted to 50 ng/ul for 454 pyrosequencing at the Max Planck Institute for Molecular Genetics, Berlin (GS FLX Titanium, Life Sciences, Roche).

### Assembly and annotation

Sequence quality assessment and trimming were performed using PRINSEQ [44] to remove short (≤ 50 bp) and low quality sequences (average phred score ≤ 20), and tail regions (phred ≤ 20, 5-base sliding window). Sequences from all six libraries that passed this step were combined to produce a single assembly using MIRA v. 3.0 [45]. Local BLASTN searches (E ≤ 10^–10^) were performed against the Silva rRNA database (LSU and SSU parc, release 108) to identify rRNA. rRNA contigs and reads were filtered with a custom BioPython script. The remaining ESTs (contigs and singletons) were compared against the NCBI non-redundant protein database (nr) using the BLASTX algorithm (E-value ≤ 10^–4^). Putative protein sequences were extracted from BLASTX output, and compared against public protein databases (KEGG, Pfam) for further functional annotation. We used the tools and resources available at the CAMERA website (https://portal.camera.calit2.net/gridsphere/gridsphere), utilizing top hits in downstream analyses. A local database (MySQL) containing normalized reads (accounting for library size differences) and annotation information was built for searches and analyses. Additional file 2 provides a schematic outline of the assembly and analysis workflow. The original sequencing data (passing quality control) are available at the NCBI Sequence Read Archive (SRA), accession number SRR575725.

### Functional annotation and statistical analysis

All orthology terms (1,207) identified from BLASTX against the KEGG protein database were mapped onto KEGG pathways using the tools available at http://www.genome.jp/kegg/ko.html. Numbers of normalized reads/library for each pathway were extracted from the local database; non-biologically meaningful pathways (e.g. human disease or other organism-specific pathways) were removed, and the data filtered for minimum KEGG terms/pathway (threshold ≥ 5) and total reads/pathway (≥ 200). This reduced the total from over 150 to a core set of 40. Library expression values (read counts) were compared using Fisher’s exact test using IDEG6 software (Identification of Differentially Expressed Genes; [46]) with Benjamini-Hochberg correction (α = 0.05; [47]): FS versus VS, MS versus VS, and MN versus VN. For graphical representation (MeV 4.8.1; [48]) data were expressed as Log_2_ (read counts Library_1_ – read counts Library_2_).

Contigs of *F. vesiculosus* were compared against the *Ectocarpus* proteome using BLASTX with an E-value cutoff of ≤ 10^-10^, and contigs matching the same *Ectocarpus* accession were combined to define an orthologous set of *F. vesiculosus* proteins (and hereafter referred to as genes). Differential expression (read counts/orthologue for each library, with a minimum cut-off of 20 reads/orthologue) between male, female and vegetative tissue was tested with Fisher’s exact tests and Benjamini-Hochberg correction, and displayed graphically as Log_2_ expression values. Distributions of expression values for vegetative-, male- and female-biased genes were compared using non-parametric Kolmogorov-Smirnov tests.

### Validation of gene expression by quantitative PCR

Material for quantitative real-time PCR (qPCR) analysis was collected at Praia Norte, Viana do Castelo, Portugal. Sampling was carried out on rising tides in July 2011 during spring and neap tidal phases (as for the EST libraries), and an additional sample was taken two days after the gamete release peak, as determined in the field (see Figure 7c in [30]). Female and male individuals were sexed by examining sectioned receptacles under the microscope, and tagged in the field prior to sampling. Individual receptacles (female and male) were collected, while apical tips (vegetative tissue) were pooled; tissue was fast frozen in liquid nitrogen and stored at -80° before further analysis. qPCR analyses were performed according to previous work on *F. vesiculosus*[49]. Briefly, total RNA was extracted from lyophilized tissue from four individual female and four individual male receptacles; for non-reproductive tissue (Veg) pools of apical tips of at least four individuals (including both female and male material) were used. RNA concentration was estimated by spectrophotometry and integrity was confirmed by running samples on a 1.2% denaturing agarose gel. First strand synthesis was performed using SuperScript III (Invitrogen) from 1 ug of total RNA in two parallel 20-μl reactions, which were then pooled. cDNAs quality were tested under standard PCR conditions (amplification conditions: 95°C for 2 min and then 35 cycles at 95°C for 30 s, 65°C for 30 s and 68°C for 20 s) and the product electrophoresed in a 2% agarose gel.

Primers for candidate genes were designed using the Primer3 web application (http://frodo.wi.mit.edu/), with a Tm of 68–70°C and an amplicon size between 100 and 250 bp. From an initial screening panel, eight candidate genes were selected for further qPCR analysis. Selection was carried out based on amplification quality and transcript expression pattern in the 454 dataset. qPCR was performed in a 96 well format, with triplicate amplifications and SYBR-green-based detection (Maxima SYBR Green/ROX, Fermentas in the Applied Biosystem step-one system). Reactions contained a 1:100 dilution of cDNA template and 0.5 μM of primers in a total volume of 20 μl. Cycle parameters were 95°C for 2 min and then 45 cycles at 95°C for 10 s and 68°C for 30 s. The amplification efficiency of each primer pair was calculated from dilution curves, using pooled cDNA from all treatment conditions as template. Relative expression was analysed with Applied Biosystems StepOne, using the ΔΔCT method corrected for amplification efficiency [50] and following [51] for applying multiple reference (housekeeping) genes: EF1d/b (elongation factor-1 B beta) and SUMO3 (small ubiquitin-like modifier). A 1:100 dilution of the pooled cDNA from all treatment conditions was used as the internal reference.

## Results

### Annotation success and library comparisons

After adaptor trimming, quality assessment and removal of rRNA, 309,281 high quality ESTs were assembled into 41,918 contigs and 3,719 singletons (Table 1). The total number of contigs with a BLASTX hit E-value ≤ 10^-4^ was 17,170 (44.7%) (Table 1). Annotation success by BLASTX was aided by the availability of the genome sequence for the brown alga *E. siliculosus*, which was the top hit species (7,591 contigs = 16.6%, corresponding to 3,853 unique protein accessions). Second, with ca. two orders of magnitude fewer accessions was the Leguminosae *Medicago truncatula* (1,136 contigs with 39 unique accessions), followed by the diatom *Thalassiosira oceanica* (850 contigs with 43 unique accessions) and *F. vesiculosus* (385 contigs with 91 unique accessions). A preliminary analysis of expression values amongst the six libraries indicated potential contamination of the female neap (FN) library by male tissue. After confirmation by qPCR tests (data not shown) the FN library was not considered further, reducing the assembly to 241,870 reads in 38,415 contigs and 2,761 singletons. Differences in expression between spring and neap tidal samples for male and vegetative tissue were low (35 and 25 genes, respectively: Additional file 3). Kolmogorov-Smirnov tests indicated that the distributions of significant DE terms did not differ between these tissue types (data not shown). Further analysis was therefore confined to the spring tide libraries.

**Table 1:** Summary statistics of 454 assembly after trimming

**Library**	**reads**	**length (bp)**	**contigs**	**N50 (bp)**	Annotated (%)*
FN**	67,411	271.8			
FS	30,316	283.4			
MN	56,978	277.3			
MS	37,717	271.1			
VN	74,765	266.9			
VS	42,094	268.3			
Total	309,281	272.2	41,918	544	17,170 (44.7)

*F *female, *M *male and *V *vegetative tissue; *N* neap tide, *S *spring tide samples.

*BLASTX; E ≤ 10^–4^.

**Possible contamination with male tissue – not analysed further.

**Figure 1:**
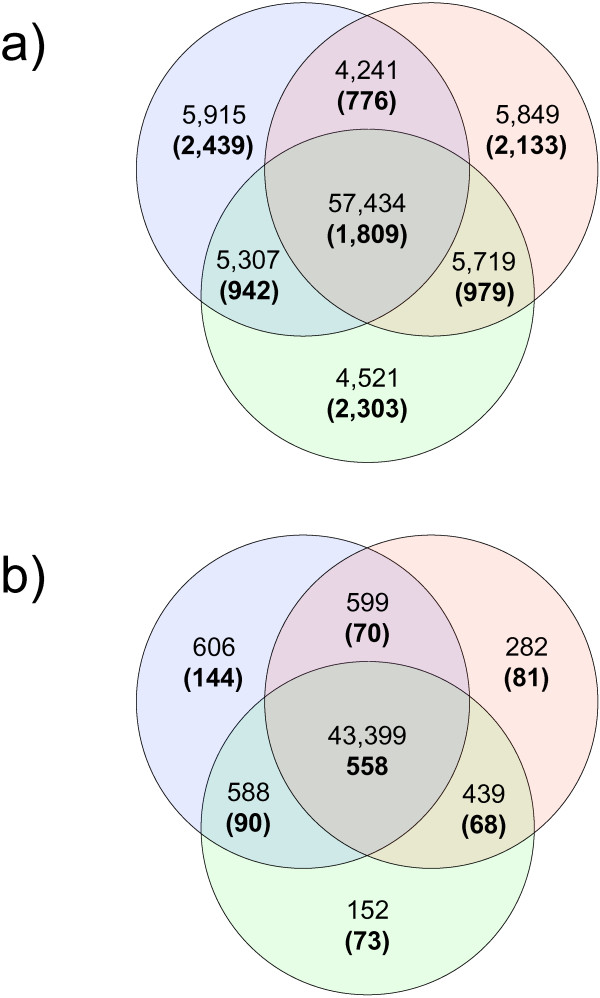
**Venn diagrams summarising the distribution of sequence reads between tissue types.** Data are from spring tide libraries for male (blue), female (pink) and vegetative tissue (green) showing **a**) normalized read counts and number of contigs (bold in parentheses) for BLASTx annotated dataset (E ≤ 10^-4^), and **b**) normalized read counts and number of Kegg orthology terms (bold in parentheses; E ≤ 10^-6^).

A relatively small subset of contigs were expressed in all tissues (1,809, 16%), but accounted for 64.5% of reads (Figure 1a). Many small contigs were assigned uniquely to individual tissues. Pairwise comparisons indicated that male and female tissues were the most differentiated, sharing the fewest contigs and reads (Figure 1a). At the functional level, a core set of 558 Kegg terms (>50%) were common to all tissues, accounting for 94% of annotated reads. Male tissue showed the greatest number of unique Kegg orthology terms (13%) and corresponding numbers of reads (Figure 1b).

### Trancriptome functional analysis

A total of 1,084 KEGG orthology terms were identified in the dataset (FS, MS, VS; see Additional file 4 for the list of KEGG terms and Additional file 5 for the list of Pfams identified), and were mapped onto 123 pathways (KEGG ko; list available as Additional file 6). ‘Translation’ was the most common functional class, represented mostly by the biochemical pathway ‘Ribosome’ (ko03010). The number of transcripts assigned to this pathway is about 7.5x higher than the second most expressed pathway, ‘Photosynthesis’ (ko00195), class ‘Metabolism’.

**Figure 2:**
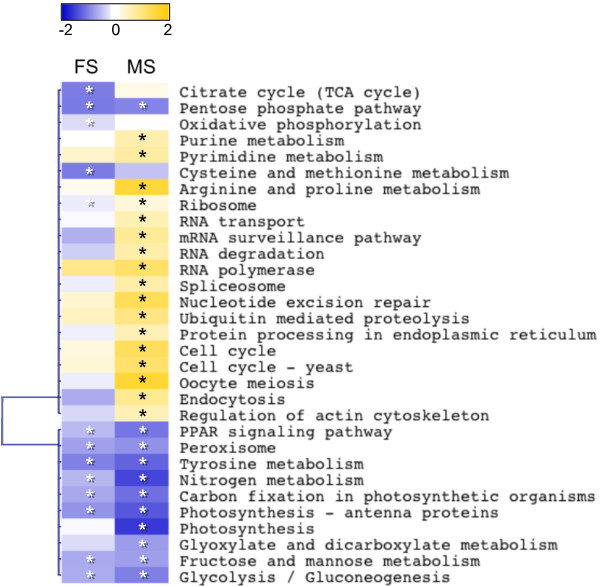
**Heatmap of differential expression in KEGG pathways between sexual and vegetative tissue. **Pixels represent Log_2_(sexual – vegetative) read counts for a pathway. Significantly different pairwise expressions were identified by Fisher’s exact tests after Benjamini-Hochberg adjustment (*P* < 0.05) between sexual and vegetative tissue collected at the Spring reproductive cycle, i.e. F vs Veg, and M vs Veg. Blue indicate under-expression and yellow over-expression of sexual relative to vegetative tissue. Asterisks indicate significant pairwise comparisons within a pathway: in white- sex under-regulated pathway compared to the vegetative tissue; in black – male- or female up-regulated pathway relative to the vegetative tissue.

Analysis of KEGG pathway expression showed that 32 of the 40 core pathways had significant differences in transcript representation (Figure 2). Relative to vegetative tissue, both female and male sexual tissues were under-represented for transcripts belonging to energy metabolism pathways, including photosynthetic carbon fixation (ko00710) and antennae proteins (ko00196). Under-expression of nitrogen metabolism (ko00910) was mainly due to fewer transcripts for carbonic anhydrases (Additional file 6), which are likely to mediate inorganic carbon acquisition in brown algae [52-54]. These differences were most obvious in the male tissue (Figure 2), also supported by the observation that transcripts for photosynthesis (ko00195; i.e. generation of reducing power in the light reactions) were under-expressed in males, but not in females. Several pathways for carbohydrate metabolism were also under-expressed in both sexes; glycolysis and gluconeogenesis (ko00010), pentose phosphate pathway (ko00030), fructose and mannose metabolism (ko00051) (Figure 2). Under-expression of transcripts for peroxisome (ko04146) and PPAR signaling (ko03320) pathways in sexual tissues was also seen. However, the main peroxisomal transcripts affected are involved in free radical detoxification and/or redox signalling (peroxiredoxin and Mpv17), rather than lipid homeostasis. Furthermore, peroxisomal, rather than chloroplast or mitochondrial targeting of these transcripts could not be confirmed. Similarly, down-regulation of the peroxisome proliferator-activated receptor (PPAR) pathway was due almost entirely to under-expression of acyl-CoA-binding protein (K08762), which binds medium- and long-chain acyl-CoA esters and has multiple cellular roles.

Male-biased expression of the ribosome pathway, as well as RNA transport (ko03013), RNA degradation (ko03018) and mRNA surveillance (ko03015), RNA polymerase (ko03020), spliceosome (ko03040), nucleotide excision repair (ko03420), protein processing in the endoplasmic reticulum (ko04141), ubiquitin mediated proteolysis (ko04120), oocyte meiosis (ko04114) and cell-cycle pathways (ko04110, ko04111) is indicative of actively differentiating and replicating cells (Figure 2, Additional file 6).

#### Comparative sex-biased gene expression

**Figure 3:**
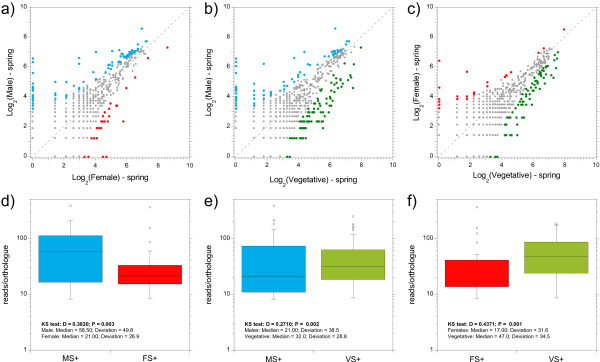
**Scatter- and boxplots comparing tissue-specific gene expression in the spring tidal phase. **Scatter plots of gene expression (as Log_2_[reads]) in **a**) female versus male tissue, **b**) vegetative versus male tissue, and **c**) vegetative versus female tissue. Each point on the plots represents a *F. vesiculosus *gene orthologue defined by mapping contigs to the *E. siliculosus *proteome using BLASTX. Only genes with ≥ 20 reads were analysed and are shown on the plots (total = 693). Points in green, red and blue indicate statistically significant over-expression in vegetative female and male tissue, respectively (Fisher’s exact tests with Benjamini-Hochberg adjustment; *P* < 0.05). Points in grey do not differ in expression between tissue types. Boxplots (lower panel) corresponding to each scatter plot show distributions of significant expression values as reads/orthologue for sets of sex- and vegetatively-biased genes: **d**) female versus male tissue, **e**) male versus vegetative tissue, and **f**) female versus vegetative tissue. MS+, FS+, and VS + = over-expressed in male, female, and vegetative spring tide libraries, respectively. The result of Kolmogorov-Smirnov tests on the distributions are summarized on each plot.

We compared the expression of male and female sex-biased genes in the spring tide phase of the reproductive cycle, when mature gametangia are present, but gamete release is minimal or null [30]. Expression in female and male sexual tissue revealed 28 female and 92 male over-expressed genes (Figure 3a). The pattern of greater male-biased expression was confirmed by comparison of sexual tissues against the vegetative background, where 27 female and 91 male over-expressed genes were identified (Figure 3b, c). More genes were under-expressed than over-expressed in females (i.e. over-expressed in the vegetative background: 76 versus 27), while numbers were similar in male tissue (89 versus 91).

The median and variance of expression value distributions of SBG were compared between female and male tissue using Kolmogorov-Smirnov tests (Figure 2d). Distributions for female and male SBG differed significantly (D = 0.382; *P* = 0.003; Figure 3d). Both the median expression (21 versus 58.5 for females and males) and the average deviation from the median (26.9 versus 49.8) were greater for male-biased genes. Differences in distributions between SBG and vegetative background were also found (*P* = 0.002 for males and *P* = 0.001 for females), and were explained mainly by elevated variance in males and reduced median in females (Figure 2e, f).

### Proteins putatively associated with sexual differentiation

After mapping of *F. vesiculosus* genes to *Ectocarpus* orthologues, a single female-specific gene was identified: a member of the mannuronan C-5-epimerase gene family. This gene catalyses the final step of alginate biosynthesis [55] (Table 2). Another female-biased gene was also identified, and related to carbohydrate modification with a likely role in cell wall biogenesis (chondroitin-D-glucuronate 5-epimerase). A gene encoding aquaporin (APQ1; K09864) that was over-expressed in female tissue in spring tide library was also over-expressed in males in the neap tide library, and was barely detectable in either vegetative tissue library (Table 2; Additional file 4).

**Table 2:** **Selected female- and male-biased *F. vesiculosus *genes, based on orthology to *E. siliculosus *proteins**

***Description***	***No. contigs***	***E. siliculosus Orthologue***	***F***	***M***	***Veg***	***KEGG***	***Pfam***	***GO***
*Female-biased:*								
Glucosylceramidase (family GH30)/ Lectin domain-like	2	CBN79893	51	4	3		PF02055; PF14200	
Mannuronan C-5-epimerase 4	1	CBN79256	24	-	-	K00771		30158
Chondroitin-glucuronate 5-epimerase	2	CBJ29234	26	4	7	K01795	PF00124	
Aquaporin PIP-type	2	CBN74001	15	3	4	K09864	PF00230	15250
Photosystem II reaction centre protein D1/psbA	66	CAV31176	930	291	807	K02703		
Imm upregulated 3	24	CBN74763	378	157	254			
*Male-biased:*								
Transcription elongation factor Elf1 like	1	CBJ25475	15	36	11		PF05129	
Touch receptor neuron protein Mec-17	2	CBJ49002	2	11	-		PF05301	
Conserved unknown protein (Josephin)	1	CBJ28700	-	22	-		PF02099	
BT1 family, putative pteridine transporter	2	CBN78031	14	49	16		PF03092	
Cystathionine gamma-lyase	2	CBN75996	-	8	-	K00810	PF01053	3962; 5843
1-phosphatidylinositol-4-phosphate 5-kinase/ radial spoke	2	CBN78497	-	5	-	K00889		16308
Ca2+/calmodulin-dependent protein kinase	1	CBN78721	-	8	-	K00908		4684; 4685
Creatine kinase	4	CBN74482	-	48	-	K00933	PF02807; PF00217	5843
Creatine kinase	4	CBN77192	-	21	-	K00933	PF00217	4111; 5842
PAS/PAC histidine kinase; putative blue light receptor	6	CBJ26132	-	30	-	K02489	PF00989	4054
F-type H + -transporting ATPase subunit delta	2	CBJ32297	2	18	4	K02134	PF02823	5843
Calmodulin	4	CBN74598	-	25	-	K02183		5842
Calmodulin	1	CBN80105	-	14	-	K02183		5842
Mitogen-activated protein kinase	3	CBJ31415	-	94	-	K04368	PF00069	5842
cAMP-dependent protein kinase regulator	4	CBJ34040	-	22	-	K04739	PF00027	5842; 8603
Proliferating cell nuclear antigen	4	CBJ25497	3	18	-	K04802	PF00705; PF02747	
Voltage dependent anion channel	3	CBN78926	5	33	12	K05862	PF01459	5842
Tubular mastigoneme-related protein (Sig)/ tenascin	3	CBJ28331	-	14	-	K06252	PF07974	
SecE/Sec61-gamma subunit protein translocation complex	17	CBN73851	85	141	103	K07342	PF00584	5842; 5843; 5874
Alpha tubulin	24	CBN77373	122	387	80	K07374	PF03953; PF00091	4111; 5842; 5843
Beta tubulin	4	CBJ26064	-	14	-	K07375	PF03953	5842
Beta tubulin	3	CBN79446	15	128	20	K07375	PF03953	5843
Dynein flagellar outer dynein arm light chain	1	CBJ48309	-	8	-	K10411		5843
Putative flagellar outer dynein arm light chain	2	CBJ27240	-	16	2	K10419	PF03259	5842
Centrin	3	CBN74045	-	29	1	K10840	PF00036	4054; 5842; 8603
Centrin	2	CBN79657	-	8	-	K10840		5842; 5843
Histone H2A	5	CBJ31989	7	74	2	K11251	PF00125	5842; 5843; 5874
Histone H2A	2	CBJ32072	7	96	12	K11251	PF00125	5842
Histone H2B	13	CBJ32076	2	31	7	K11252	PF00125	5842; 5843; 5874
Histone H3	5	CBN79887	32	135	73	K11253	PF00125	5842; 5843; 5874
AP-2 complex subunit sigma-1	1	CBN77646	2	14	1	K11827	PF01217	5843
Herpes virus major outer envelope glycoprotein	5	CBN77192	-	81	-		PF05109	

The table shows the number of *F. vesiculosus *contigs mapping (BLASTX, E-value ≤ 10^-10^) to an *E. siliculosus *protein; descriptions are based on the accession and/or annotations in the KEGG, Pfam and GO databases. Expression is shown for Spring Female (F), Male (M) and Vegetative (Veg) libraries (as normalized reads; see Results for more details).

In contrast to females, many more male-biased or male-specific genes were identified (Table 2). Amongst male-specific terms, signalling-related genes were conspicuously over-represented. Most striking was a mitogen activated protein kinase (MAP2K; K04368), a cAMP-dependent protein kinase regulator (PRKAR; K04739), a PAS-PAC histidine kinase and putative blue-light photoreceptor, calmodulin genes and a Ca^2+^/calmodulin-dependent protein kinase (CaMK; K00908). This provides a general picture of male tissue as very active in signalling for a potentially diverse range of cellular processes. The over-expression of dynein-related molecular motor proteins (K10410, K10411), tenascin-like *Sig* (sexually induced) genes and creatine kinases involved in energy storage and release are likely related to specific flagella localization and functions. We also identified male-biased genes for histones, centrin, as well as α- and β-tubulins (Table 2).

### Gene expression validation by qPCR

**Figure 4:**
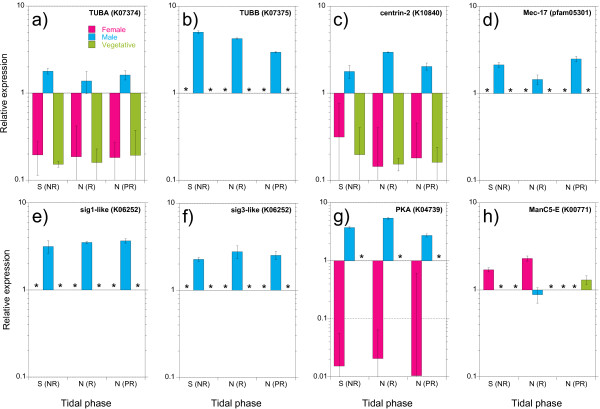
**RT-qPCR analysis of gene expression for selected male- and female-biased genes.** Values are relative expression (fold-change plotted on a Log scale, relative to a pool of all samples normalized to housekeepers EFd/b1 and SUMO3) for female (red), male (blue) and vegetative (green) tissue collected from the field during spring tide (S), and neap (N) tides. Genes were annotated based on homology to *Ectocarpus siliculosus *proteins, KEGG orthology, and/or Pfam databases; **a**) TUBA (α-tubulin), **b**) TUBB (β-tubulin), **c**) centrin-2, **d**) Mec-17-like protein (mechanosensitive touch receptor), **e**) *Sig1* (diatom sex-induced gene homologue), **f**) *Sig3*, **g**) PKA (protein kinase A), and **h**) ManC5-E (mannuronan C5 epimerase). Asterisks indicate expression values below the cut-off for detection. (NR) = no gamete release, (R) = gamete release, and (PR) = post gamete release in the field. See Results for descriptions of gene targets.

Quantitative RT-PCR analysis of independent samples of sexual and vegetative tissues confirmed the results obtained by pyrosequencing. Seven male-biased and one female-biased transcripts were selected after tests for amplification efficiency. The specific contig and primer sequences are given in Additional file 7. In agreement with pyrosequencing results, alpha-tubulin (TUBA) and centrin were confirmed as male-biased, with low but detectable expression in non-male tissue (Figure 4a,c), while a beta-tubulin isoform, a Mec-17 domain protein (pfam05301; touch receptor protein), and 2 tenascin-like proteins homologous to sexually induced (*Sig*) genes 1 and 3 from the diatom *Thalassiosira weissflogii* were male-specific (Figure 4d,e,f). A cAMP-dependent protein kinase regulatory subunit (PKA), which was only detected in male libraries, was highly male-biased in qPCR tests, with > 20 fold lower expression in females and below detection limits in vegetative tissue (Figure 4g). Variation in expression within the tidal cycle was less clear, although increased expression in males at neap tide for some transcripts (centrin, PKA) agrees with pyrosequencing results. Finally, the female-biased expression of a mannuronan C-5-epimerase was also confirmed (Figure 4h). Interestingly, expression of this transcript was not detected in the post-neap sample (i.e., following gamete release) (Figure 4h), suggesting that it may be localized to mature oogonia.

## Discussion

This study provides the first transcriptomic analysis of expression variation in reproductive tissues for a brown alga during natural reproductive cycles. We show that primary energy and carbohydrate metabolic pathways are under-represented in sexual (male and female) tissues. Differentiation is most clearly apparent in male tissue (Figure 2). At the same time, pathways for genetic information processing and cell-cycle related processes were over-represented in males. Expression analysis of protein annotations confirmed the view that male-biased expression involved greater numbers of gene products, and that variance in expression was greater for male- than for female-biased genes. Moreover, specific transcripts were found in male receptacles that were not detected in females, consistent with sperm-specific developmental and signaling pathways. Finally, our analyses (confirmed by RT-qPCR) revealed several strongly sex-biased or sex-specific transcripts that are potential targets for expression and/or sequence analysis for studying ecological and evolutionary processes in recently diverged sister species of *Fucus*.

### Pathway analysis reflects sex-specific variation in metabolism and cell proliferation

Primary metabolism for energy and carbohydrate pathways differed between the sexes and between sexual and vegetative tissue, presumably reflecting functional constraints and trade-offs associated with different developmental programs and sexual phenotype. Receptacle photosynthetic rates are reported to be ca. 50% lower than in non-reproductive tissue in *F. serratus*[56], although no information on sex-specific variation is available. At the transcriptional level, male under-expression of photosynthesis-related pathways exceeded that in females, particularly for light energy capture (antennae proteins) and photosynthetic electron transport (neither of which differed between female and vegetative tissue at spring tide). This could reflect greater female investment in gamete provisioning.

While respiratory pathways were under-expressed in sexual tissue generally, neither oxidative phosphorylation nor the citrate (TCA) cycle in males were significantly different from vegetative tissue during the spring tide phase of reproduction, the period when energy expenditure in spermatogenesis, and the maturation of anteridia for release on the following neap tide cycle should be greatest. The fucoid sperm is the only motile phase of the life cycle, showing a heterokont biflagellated cell pattern [57]. Sperm flagella have two main functions requiring high energy consumption: swimming and mediation of gamete-gamete interactions during fertilization (via the anterior mastigoneme-bearing flagellum; [58]). Transcripts for creatine and arginine kinases (arginine and proline metabolism pathway; Additional file 4), were also highly expressed in males, and virtually absent from other tissues. Phosphocreatine and phosphoarginine are storage molecules forming an energy reservoir from which ATP can be rapidly generated to replenish cellular energy balance in highly active cells.

The estimated sperm-egg ratio is 400:1 in *F. vesiculosus*[23], so elevated levels of cell cycle activity and associated processes during gametogenesis in male tissue may arise simply because a greater number of cells (and proportion of mRNA) in the tissue sample are affected. Cell cycle and oocyte meiosis pathways were over-expressed uniquely in males, and were accompanied by male over-expression of pathways involved in transcription (RNA polymerase, spliceosome), translation (ribosome, mRNA surveillance), protein folding, sorting and degradation (ubiquitin-mediated proteolysis, protein processing in the endoplasmic reticulum, RNA degradation), and replication and repair (nucleotide excision and repair). Overall, pathway analysis strongly reflects higher replicative activity in male relative to female reproductive tissue.

### Patterns of sex-biased gene expression

High throughput sequencing technologies have increased our focus on sex-biased gene expression with the realization that a large proportion of the transcriptome in many organisms shows distinct patterns of male and female expression [4,16,59-61]. Sexual dimorphism occurs against an almost identical genomic background, implying that the majority of sexually dimorphic traits may experience differential, or even conflicting, selection pressure in males and females [4]. Several studies have identified demasculinization (under-representation of male-biased genes) and/or femininization (greater numbers of female-biased genes) on X chromosomes in organisms with heterogametic male (XY) chromosomes ([4,19,62-66]; see also [15] for ZW sex determination in birds). However, the evolutionary dynamics of demasculinization may be complex, with evidence in *Drosophila* that recent male-biased genes may initially accumulate on the X chromosome and be lost over time [67].

This is the first dataset to our knowledge that can begin to address the question of sex-biased gene expression in brown algae, an independent multicellular lineage [20]. Despite a relatively low sequencing depth, we found more genes over-expressed in males than in females (ca. 13% versus 4% of the total), and that both the mean and variance of expression were significantly greater in males. We currently know little about sex determination in brown algae other than that it is genetically based [68], although the recent discovery of a non-recombining chromosomal locus in *Ectocarpus* promises progress in this area (SM Coelho and JM Cock, pers commun; [69]). Since most evidence from the literature suggests greater sex-biased expression in the heterogametic sex (males in *Drosophila*, *Silene*, females in birds), it will be interesting to further study sex-determination and the occurrence or not of heterogametic sex in *Fucus.*

### Sex-biased gene expression in females and pleiotropic constraint

Relatively few over-expressed female annotations were identified in our dataset. Overall, our data are consistent with widespread pleiotropic effects in female-effect genes [1,17,18,59].

The three clearest examples of female-biased genes are all carbohydrate-modifying enzymes. Two were epimerases - mannuronan C-5-epimerase and chondroitin-D-glucuronate 5-epimerase (COG0451) - with roles in cell wall biogenesis and/or modification. Mannuronan C-5-epimerase is a multigene family in brown algae [55], performing the final step in alginic acid biosynthesis that is critical for the formation of the alginate-rich brown algal cell wall. Of the 13 contigs in our dataset with top Blast hits to 12 predicted mannuronan C-5-epimerase proteins in *Ectocarpus*, three were female-unique, and one was expressed at a relatively higher level in female reproductive tissue. A third transcript encodes a homologue to an *Ectocarpus* glucosylceramidase. This transcript(s) contained both an O-glycosyl hydrolase (hydrolysis of glycosidic bonds) and a lectin-like (carbohydrate-binding) domain. It seems unlikely that this gene product participates directly in the fertilization process, as the presence of lectin domains on female gamete recognition proteins runs contrary to what is known about gamete recognition in *Fucus*, or invertebrates, where sperm-localized glycoproteins bind carbohydrate ligands on the egg [31,41,42,70]. The functional significance of this gene therefore remains to be seen.

Gametangial expulsion from fucoid receptacles is a rapid (min) process [34], mediated by osmotic adjustments associated with periods of K^+^ and Cl^-^ efflux from oogonia into the conceptacle extra-cellular matrix (ECM) [36]. Subsequent swelling of the ECM and/or expansion of anchoring gametangial stalk cells may be key events driving gametangial release. We identified a sex-biased (FS and MN) aquaporin-1 that could potentially play a key role in controlling rapid movements of water between cells and the ECM. Although previous work with inhibitors provided evidence for the involvement of K^+^ uptake, slow-type anion channels, and tyrosine phosphorylation in gamete expulsion [34], our transcript profiles failed to reveal candidate genes with potential roles in these processes in sexual tissue.

### Male-biased and male-specific patterns of gene expression: putative sperm signal transduction and flagella function

The number of male-biased and -unique annotations confirms male reproductive tissue as being highly differentiated compared with either female or vegetative tissue. The majority of these gene products are likely components of the flagella (e.g., tubulin, dynein), or flagella basal bodies (e.g. centrin; [71]). In addition, a number of genes with known functions in signal perception and transduction and/or cytoskeletal regulation were male-specific. A phototropin-like blue light receptor was uniquely expressed in males, with homology to an *Ectocarpus* PAC/PAS sensor histidine kinase and green algal phototropins. Fucoid sperm are negatively phototactic, and this gene is a prime candidate for the sperm photoreceptor. The absence of any transcript for a putative blue-light receptor in female tissue (including putative opsin-like proteins; [72]) is curious, since photopolarization by blue-light is one of the first axis-forming responses of the zygote after fertilization [73,74]. It remains possible however, that expression of some female genes at the neap tide (gamete-release) phase of the reproductive cycle are under-represented in the spring tide library, when numbers of mature oogonia may be lower [30,34].

Eukaryotic cilia and flagella use both cyclic adenosine monophosphate (cAMP) and calcium as second messengers to regulate the beating of dynein-driven microtubules in the axomene [75]. We detected a male-specific cAMP-dependent protein kinase (PKA) regulatory subunit. PKA is known to play a key role in mammalian sperm capacitation [76] via the cAMP/PKA signaling pathway. cAMP-dependent phosphorylation of flagella proteins, particularly axonemal dynein, is essential for the initiation and maintenance of mammalian sperm motility, with PKA the likely downstream kinase responsible for phosphorylation [77,78]. Therefore, although we were unable to confirm the presence of adenylate cyclase or potential PKA-anchoring proteins in our dataset, the male-specific expression of PKA regulatory subunit hints at shared components in sperm signalling pathways in brown algal and mammalian systems.

Male transcriptome data revealed the expression of a specific CaMK, a male-specific calmodulin isoform, and a dynein-light chain (both the latter with CaM EF-hand Ca^2+^-binding domains). Calcium and Ca^2+^ signalling play a central role in flagella function and sperm motility from mammals to green algae [79,80], with evidence for the involvement of calmodulin (CaM) and Ca^2+^/CaM-dependent kinases (CaMK) that modulate dynein activity in the axoneme [81-83]. The identification of a male-specific 1-phosphatidylinositol-4-phosphate 5-kinase (PIP5K) annotated as a axonemal radial spoke protein in *Ectocarpus* may further indicate a link between the phosphatidylinositol signalling pathway, releasing Ca^2+^ from intracellular stores, with Ca^2+^ induced modulation of flagella activity in sperm.

Finally, a highly over-expressed mitogen activated protein kinase kinase (MAPKK) was identified in male reproductive tissue. The MAP kinase pathway has many important roles mainly in the transduction of extracellular signals, particularly in plant stress signalling pathways [84], but also more generally in the control of cell growth, death, proliferation and differentiation. In the context of male-specific expression, MAP kinases are implicated in the control of flagella development in Chlamydomonas [85].

### Male-specific candidate genes for gamete-gamete interactions

Homologues to the sexually induced protein (*Sig*) family, first identified in *Thalassiosira weissflogii* by Armbrust [86], showed male-specific expression in *Fucus vesiculosus*. Based on homology with *Ectocarpus* predicted protein sequences, four independent transcripts were identified, with sequence similarities to *T. weissflogii Sig*1-3. The most highly expressed member was *Sig*1-like and contained cysteine-rich epidermal growth factor (EGF)-like domains and similarity to extracellular tenascin-like glycoproteins involved in vertebrate cell adhesion [87,88]. Interestingly, some *Ectocarpus* homologues are annotated as tubular mastigoneme proteins of the anterior flagellum. Mastigonemes are tubular hairs present on the anterior flagellum of heterokonts (but lacking in diatoms) that play an important role in establishing sexual contact in *Ectocarpus*[58]. A second group of five male-specific contigs homologous to *Ectocarpus* CBN79192, and with limited homology to Herpes virus major outer envelope glycoprotein (Herpes_BLLF1; Pfam05109) could also be involved in cell-cell interactions.

After earlier work established some of the characteristics of sperm-egg recognition in *Fucus*[41,42,89], further progress has slowed due mainly to a lack of genomic information. Candidate genes for potential gamete recognition proteins will stimulate further molecular analysis (e.g. the *Sig* gene family) in the context of speciation, one of the primary motivations for this study. Diversification within the genus *Fucus* began fairly recently, and is ongoing [21,90]. While barriers to interspecific fertilization between more distantly-related members operate under normal conditions [91], historical and/or ongoing hybridization has been inferred in several studies [22-25,92]. Species complexes where barriers to hybridization range from ecological factors [24,30] to (potential) divergence in gamete recognition proteins promise insight into the evolutionary processes underlying species divergence.

## Conclusions

Our study compares gender-specific sexual and vegetative transcriptional profiles in an oogamous brown alga, providing the first large scale transcriptomic dataset for fucoid seaweeds, and increases the number of annotated mRNA sequences in *F. vesiculosus* to over 17,000 from the several hundred available previously [49]*.* Matches to over 3,800 unique protein accessions were identified against the only sequenced brown algal genome (*Ectocarpus siliculosus*). Transcriptomic datasets like this will be a useful contribution for future annotation of the *F. vesiculosus* genome (http://www.cemeb.science.gu.se/research/imago-marine-genome-projects/).

Male, but not female, reproductive tissue over-expressed cell cycle and meiotic pathways relative to vegetative tissue, as well as several genetic information processing pathways that reflect increased replicative activity associated with sperm production. At the same time, energy and carbon metabolism pathways were down-regulated in reproductive tissue, again most strongly in males. As suggested by these results at the systems-level, orthology-based analysis showed that 1) the number of sex-biased genes was ca. three-fold higher in male compared with female reproductive tissue, and that 2) the average expression level of male-biased genes was greater than female-biased genes. These observations support the contention that males express a wide array of genes regulating male fitness, and agree with similar observations in metazoan models, where greater transcriptional rates in the male germline, functional (pleiotropic) constraint in females, or male competition have been suggested as possible drivers [4,17].

A major aim was to identify candidate sex-biased genes with potential sexual and reproductive functions. Female-biased candidate genes were limited to carbohydrate-modifying enzymes with likely roles in cell wall/matrix modification, at least at the sequencing depth employed here. In contrast, a variety of male-specific transcripts were identified related to development, signalling and signal perception, and potential flagella-localized proteins (potential candidate gamete-recognition proteins). Taken together, this study provides a valuable addition to the scarce genomic resources available in brown algae, and a starting point for future studies of possible mechanisms underlying prezygotic isolation in this system, a first step contributing to species divergence.

## Abbreviations

SBG: Sex-biased genes; genes expressed in both sexual reproductive tissues; F: Female tissue/receptacles; M: Male tissue/receptacles; Veg: Vegetative tissue/apical tips; N: Neap tidal phase (sampling); S: Spring tidal phase (sampling); ECM: Conceptacle extra-cellular matrix; Male-biased genes: Genes up-regulated in the male tissue; Female-biased genes: Genes up-regulated in the female tissue; Male-unique: Genes expressed in the male tissue exclusively, with no identified reads in remaining tissue types analysed; Female-unique: Genes expressed in the female tissue exclusively.

## Competing interests

The authors declare that they have no competing interests.

## Authors’ contributions

GAP designed the study. GAP and CFM sampled material in the field, CFM prepared cDNA libraries for pyrosequencing, MJFM carried out the RT-qPCR experiments. MJFM and GAP analysed the data and performed the bioinformatic analyses. MJFM and GAP drafted the manuscript and all authors contributed to editing the final version. All authors read and approved the final manuscript.

## Supplementary Material

Additional file 1 ***Fucus vesiculosus. ***Image of an *F vesiculosus *specimen with indication of the reproductive and non-reproductive tips, line drawing of female conceptacle and contents. Click here for file

Additional file 2 **Schematic outline of sequence processing and analysis workflow.** Flow-diagram showing the workflow for sequence quality assessment, assembly, annotation, and simple database structure. Click here for file

Additional file 3 **Scatter plots comparing gene expression at minimum (spring tide) and maximum (neap tide) reproductive phases. **Each point represents a *F. vesiculosus *gene orthologue defined by mapping contigs to the *E. siliculosus *proteome using BLASTX. Genes with statistically significant differences in expression between reproductive phases in a) vegetative tissue are shown by green points, and b) in male tissue by blue points (Fisher’s exact tests with Benjamini-Hochberg adjustment; P < 0.05). Grey points indicate genes with no significant variation in expression with respect to tidal phase. Click here for file

Additional file 4 **Summary of KEGG annotation data per library. **Summary of KEGG annotation data for Female (F), Male (M) and Vegetative (Veg) tissue; S = spring tide; N = neap tide. Values are normalized read counts; total normalized reads for the assembly are given in the last column. Click here for file

Additional file 5 **Summary of Pfam annotation data per library. **Summary of Pfam annotation data for Female (F) Male (M) and Vegetative (Veg) tissue; S = spring tide; N = neap tide. Values are normalized read counts; total normalized reads for the assembly are given in the last column. Click here for file

Additional file 6 **Summary of KEGG pathway analysis. **Summary of KEGG pathway data for Female (Fem), Male (M) and Vegetative (Veg) tissue; S = spring tide; N = neap tide. Click here for file

Additional file 7 **Primer sequences for qPCR analysis. **Primer sequence, annotation information (NCBI accession number of *Ectocarpus siliceous *orthologous) and sequence description, and application size for the eight target genes studied and the two reference genes (housekeepers). Click here for file
