# Supplementary material for: Sex-biased gene expression in the brown alga Fucus vesiculosus
Source: BMC Genomics. 2013 May 1;14:294. doi: 10.1186/1471-2164-14-294 (PMC3652789; doi:10.1186/1471-2164-14-294)
Supplement: Additional file 1 — Fucus vesiculosus. Image of an F vesiculosus specimen with indication of the reproductive and non-reproductive tips, line drawing of female conceptacle and contents. [file 1471-2164-14-294-S1.pdf]

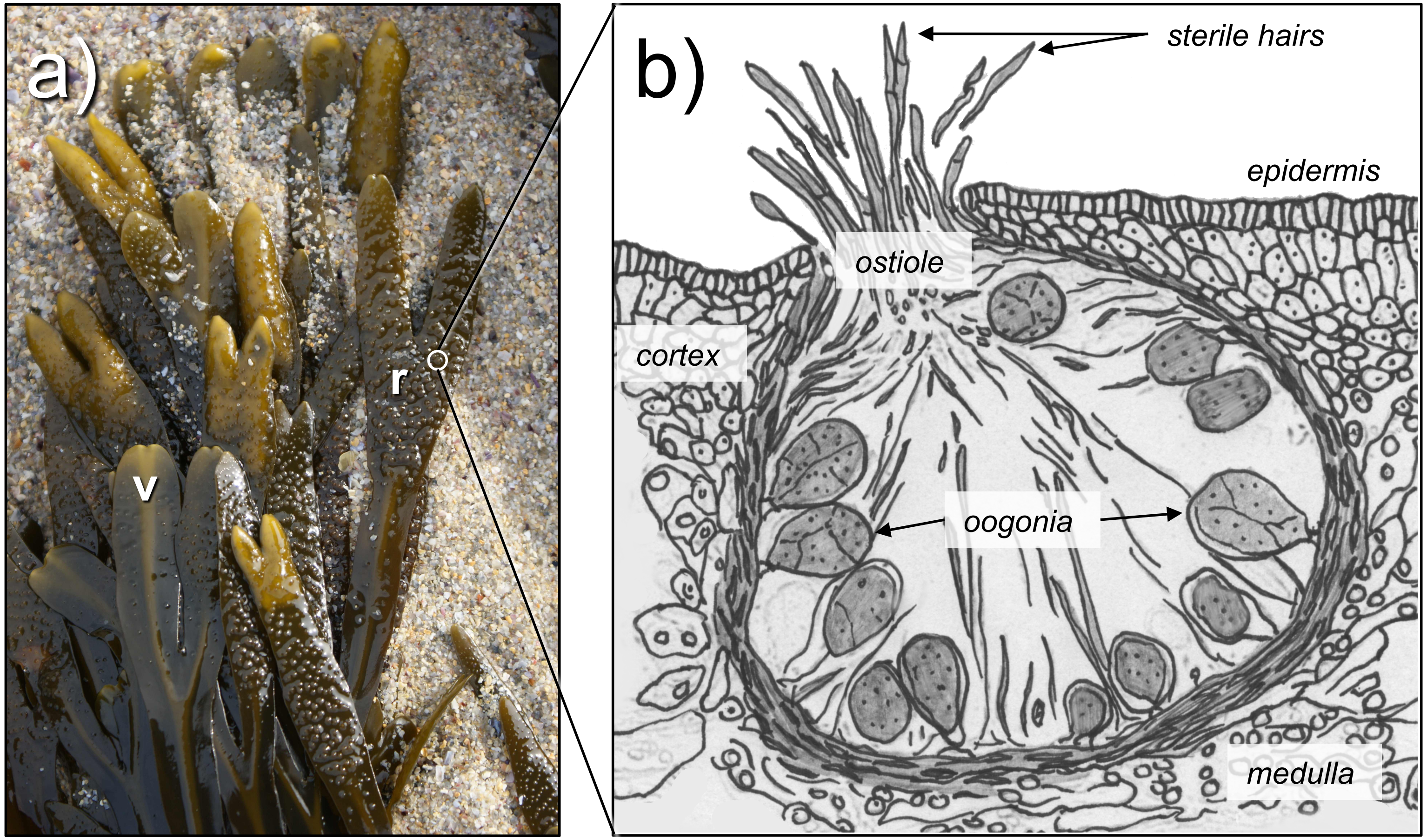

Additional File 1. a) Adult individual of *F. vesiculosus* indicating apical vegetative (v) and reproductive tissue (receptacle, r). b) Cross-sectional drawing of a female conceptacle. Oogonia are expelled during gamete release through the ostiole to the external seawater, guided by sterile hairs. Oogonia breakdown in seawater to release 8 eggs.
