# Supplementary material for: Sex-biased gene expression in the brown alga Fucus vesiculosus
Source: BMC Genomics. 2013 May 1;14:294. doi: 10.1186/1471-2164-14-294 (PMC3652789; doi:10.1186/1471-2164-14-294)
Supplement: Additional file 2 — Schematic outline of sequence processing and analysis workflow. Flow-diagram showing the workflow for sequence quality assessment, assembly, annotation, and simple database structure. [file 1471-2164-14-294-S2.pdf]

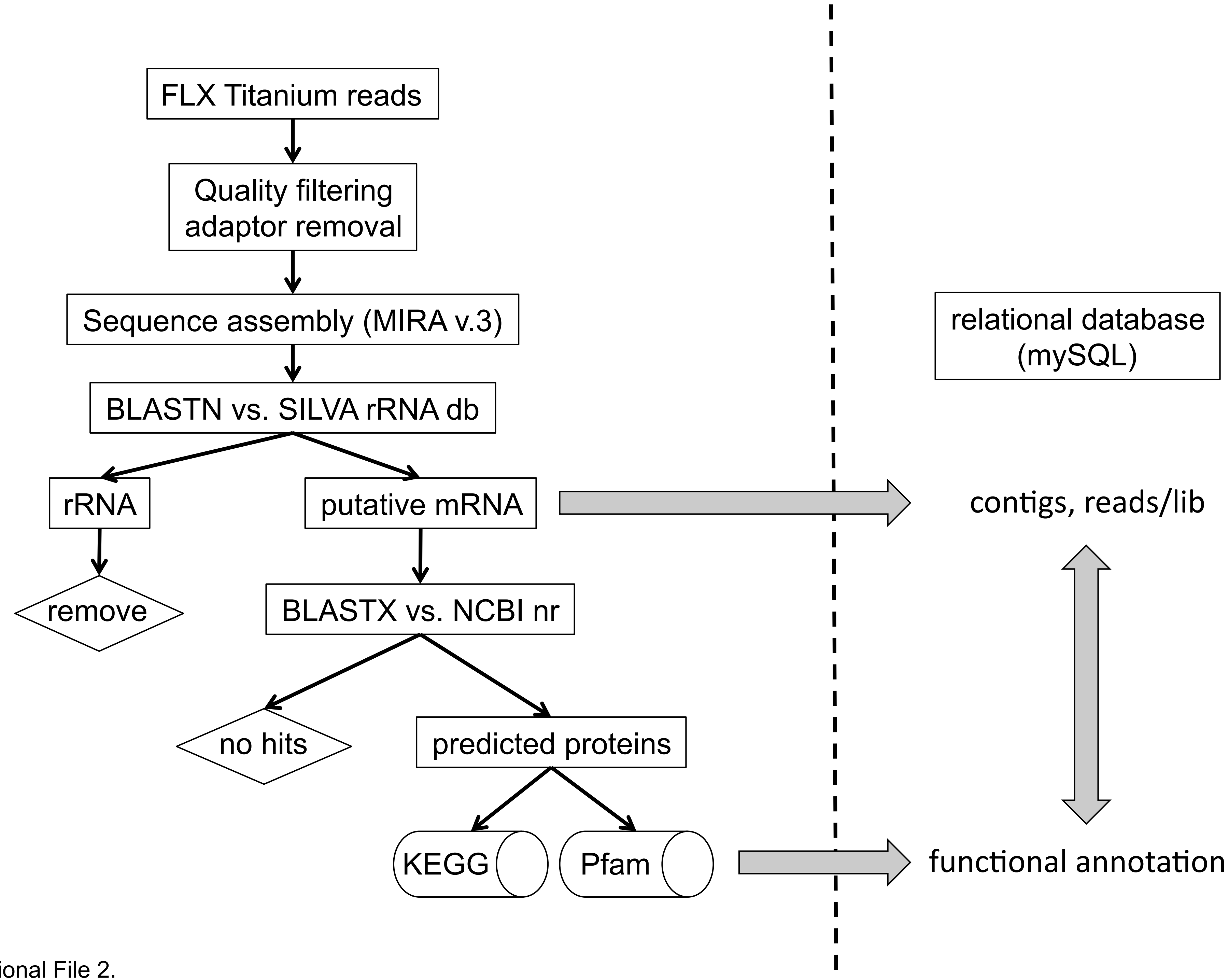

Additional File 2.
Flow-diagram showing the workflow for sequence quality assessment, assembly, annotation, and simple database structure.
