# Supplementary material for: Sex-biased gene expression in the brown alga Fucus vesiculosus
Source: BMC Genomics. 2013 May 1;14:294. doi: 10.1186/1471-2164-14-294 (PMC3652789; doi:10.1186/1471-2164-14-294)
Supplement: Additional file 3 — Scatter plots comparing gene expression at minimum (spring tide) and maximum (neap tide) reproductive phases. Each point represents a F. vesiculosus gene orthologue defined by mapping contigs to the E. siliculosus proteome using BLASTX. Genes with statistically significant differences in expression between reproductive phases in a) vegetative tissue are shown by green points, and b) in male tissue by blue points (Fisher’s exact tests with Benjamini-Hochberg adjustment; P < 0.05). Grey points indicate genes with no significant variation in expression with respect to tidal phase. [file 1471-2164-14-294-S3.pdf]

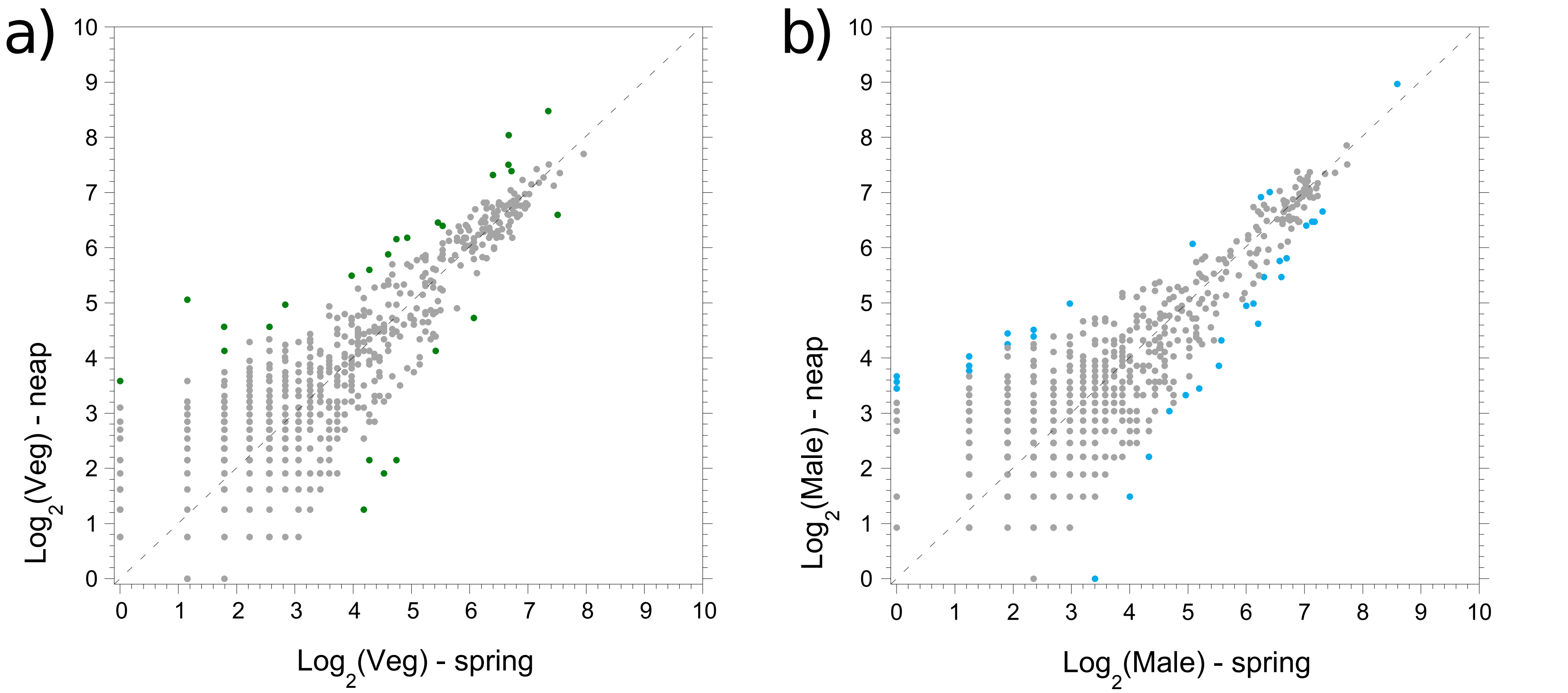

Additional File 3. Scatter plot comparing gene expression at minimum (spring tide) and maximum (neap tide) reproductive phases. Each point represents a *F. vesiculosus* gene orthologue defined by mapping contigs to the *Ectocarpus siliculosus* proteome using BLASTX. Genes with statistically significant differences in expression between reproductive phases in a) vegetative tissue are shown by green points, and b) in male tissue by blue points (Fisher's exact tests with Benjamini-Hochberg adjustment; $P \leq 0.05$). Grey points indicate genes with no significant differences in expression between tidal phases.
